# Sudden Death during Labor

**Published:** 1890-11

**Authors:** L. H. Hardy

**Affiliations:** Paige, Texas


For Daniel's Texas Medical Journal.

### REPORT OF A CASE OF SUDDEN DEATH IN LABOR.

BY L. H. HARDY, M. D., PAIGE, TEXAS.

Read before the Austin District Medical Society, March, 1890.

WAS called to see Mrs. B. on the night of November 14, 1888. Primapara age twenty-two years. Married eleven months. Health during pregnancy very good. I reached the house, in the country about three miles from home, about 11 o'clock, and found the patient suffering with an occasional premonitory pain. The night being cool, I did not go immediately to the bedside but warmed myself half an hour. On examination found the os sufficiently dilated to admit my finger, by the use of which I tried to induce better pains. Finding the condition all right as far as I could tell, and seeing no necessity of remaining by the bedside, I resumed my seat by the fire. At short intervals I would repeat examination. making efforts to bring about better pains.

After waiting two or three hours for the pains to increase and finding but slight dilation, I gave eight or ten grains of quinine, as I frequently do, which I have found effectual in many cases. Pains coming on more frequently, but of short duration, I began the use of chloroform, hoping thereby to make the pains more bearable and to relax the muscular system. All the while I was trying to encourage the lady to be patient, and assuring her that all would be well in the course of time. Finding that labor was not progressing as satsifactorily as desired and seeing patient's fortitude failing, I started a messenger after instruments and told him to bring Dr. T. W. Styles.

Up to this time nothing in the condition of the patient gave rise to the least suspicion of impending mischief. When all at once an intense and horrible dyspnoea came on. She gasped and struggled for breath, endeavoring in vain to get more air. Muscles of face and thorax were violently agitated in the attempt to oxygenise the blood. The lips and face were deeply cyanosed.

Her cries were the most heartrending I ever heard, asking for help and saying that she was dying. I immediately saw that death was inevitable unless something could be done. I ordered whiskey given and mustard applied, and as soon as I could I admistered digitalis and belladonna hypodermically. In less than fifteen minutes after I first noticed the condition of the patient she had breathed her last.

During my last examination I told patient her's was a tedious case, but I hoped that pains would soon change and that she would soon be delivered.

Such, gentlemen, is the only case of sudden death ever witnessed by myself, and I trust to never have the same sad experience again, for I have felt the effect of the loss of that case, especially in that neighborhood.

The question arises: What was the cause of death in the case just related? Could it have been shock? Rupture of heart? Bursting of aneurism? Or was there an obstruction of pulmonary artery? I am inclined to think the latter. No autopsy was had.

[In addition to the above queries as to the cause of death, it might be suggested that rupture of the uterus is a very frequent cause of sudden death in labor. It is to be regretted that post mortem examinations are not more common in such cases. Dr. Hardy is certainly not gulty of any mismanagement of the case, for such unfortunate deaths are inevitable and may happen in the practice of any physician.—B.]

